# Interactions Between Environment and Genetic Diversity in Perennial Grass Phenology: A Review of Processes at Plant Scale and Modeling

**DOI:** 10.3389/fpls.2021.672156

**Published:** 2021-11-16

**Authors:** Simon Rouet, Romain Barillot, Denis Leclercq, Marie-Hélène Bernicot, Didier Combes, Abraham Escobar-Gutiérrez, Jean-Louis Durand

**Affiliations:** ^1^INRAE, URP3F, Lusignan, France; ^2^Groupe d’Etude et de Contrôle des Variétés Et des Semences (GEVES), Lusignan, France; ^3^Groupe d’Etude et de Contrôle des Variétés Et des Semences (GEVES), Dijon, France

**Keywords:** climate change, grasslands, heading date, perennial grasses, phenological models, phenology, photoperiod, temperature

## Abstract

In perennial grasses, the reproductive development consists of major phenological stages which highly determine the seasonal variations of grassland biomass production in terms of quantity and quality. The reproductive development is regulated by climatic conditions through complex interactions subjected to high genetic diversity. Understanding these interactions and their impact on plant development and growth is essential to optimize grassland management and identify the potential consequences of climate change. Here, we review the main stages of reproductive development, from floral induction to heading, i.e., spike emergence, considering the effect of the environmental conditions and the genetic diversity observed in perennial grasses. We first describe the determinants and consequences of reproductive development at individual tiller scale before examining the interactions between plant tillers and their impact on grassland perenniality. Then, we review the available grassland models through their ability to account for the complexity of reproductive development and genetic × environmental interactions. This review shows that (1) The reproductive development of perennial grasses is characterized by a large intraspecific diversity which has the same order of magnitude as the diversity observed between species or environmental conditions. (2) The reproductive development is determined by complex interactions between the processes of floral induction and morphogenesis of the tiller. (3) The perenniality of a plant is dependent on the reproductive behavior of each tiller. (4) Published models only partly explain the complex interactions between morphogenesis and climate on reproductive development. (5) Introducing more explicitly the underlying processes involved in reproductive development in models would improve our ability to anticipate grassland behavior in future growth conditions.

## Introduction

Grasslands are one of the most widespread terrestrial ecosystems, covering around 52.5 million km^2^^1^ and constituting the basis of many agrosystems. They provide multiple ecosystemic services such as forage production for herbivores, preservation of water quality, erosion control, maintenance of biodiversity and carbon storage (Huyghe et al., 2012; Martin et al., 2020). Despite the importance of grasslands in livestock farming, the quantity and quality of forage are often sub-optimal (Boval et al., 2015; Doole and Romera, 2015). This is partly explained by a mismatch between the dynamics of biomass production and the harvesting dates (mowing or grazing). Improving our capacity to predict the annual fluctuation of biomass quantity and quality is therefore crucial to increase the efficiency of these major agrosystems. Natural and cultivated grassland formations are dominated by *Poaceae* species which, botanically, have very similar reproductive developments (Thomas, 1980).

One of the main components of the annual dynamics in grassland productivity is the reproductive development which encompasses a set of processes ranging from floral induction to seed production. The start of the reproductive development is a major phenological event occurring in spring in temperate climate areas and a key episode for grassland management. Firstly, it is often associated with a peak in biomass production due to a concomitant increase of leaf growth rate (Parsons and Robson, 1980). Secondly, following the start of the reproductive development, internodes of grasses begin to elongate, which decreases the biomass quality for ruminants (Chapman et al., 2014). Finally, the start of the reproductive development also affects the perenniality of grasslands, as reproductive tillers die after heading (spike emergence from the tiller’s pseudostem) and seed dispersal (Barre et al., 2017). Also, the reproductive development of perennial grasses allows the genetic evolution of grassland species and affects other ecosystemic services provided by grasslands, such as the hosting of beneficials and pathogens and the rate of leaf turnover, which alters litter quality.

**FIGURE 1:**
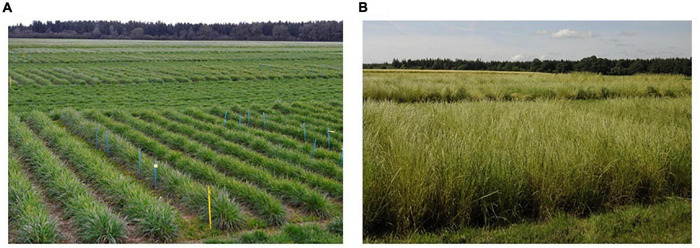
Effect of the reproductive development on the above-ground morphology in *Lolium perenne*. **(A)** Cultivars of *Lolium perenne* at vegetative state on March 20th 2018. **(B)** Same plants at reproductive state on June 8th 2018. The experiment is conducted by GEVES in Lusignan (France).

**FIGURE 2:**
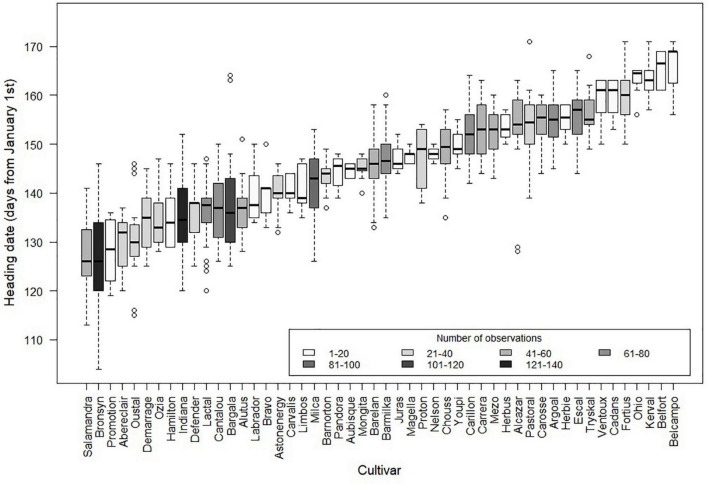
Heading dates of 50 commercial cultivars of *Lolium perenne* grown for 17 years in seven French locations with contrasted climatic conditions. Heading date is measured as the first date when at least 10 tillers reach heading per linear meter of plants sown during the previous spring. Boxplots represent the variability of heading date between year-location combinations for each cultivar (plot in the style of Tuckey: –1.5*IQR, 2nd quartile, median, 3rd quartile, + 1.5*IQR). The grayscale represents the number of observations per cultivar.

Despite the importance of the reproductive development on grassland functioning and management, there is still a significant knowledge gap that prevents predicting the occurrence of reproductive stages for a wide range of environments and genotypes. The simulation of the reproductive development in grasslands integrating environment and genetics faces several challenges which should be addressed in numerical models. Firstly, the role of environmental factors must be identified and quantified in order to account for various climatic conditions and inter-annual variations. This aspect has become even more crucial in the context of climate change. For a given region, climate change may result in combinations of photoperiod, temperature and water availability never encountered before, which could significantly affect the phenological events of local perennial grass species and cultivars. Over the last 20 years already, Vuffray et al. (2016) have observed earlier heading dates and a more frequent alternation between very early and very late headings. Such changes would combine with the effects of projected modifications in water balance and atmospheric CO_2_ concentration. Secondly, the diversity of phenotypes observed among cultivars and species must be accounted for, as grasslands are usually mixtures of species, each of them exhibiting an intraspecific diversity. These genetic diversities increase the complexity for the prediction of reproductive development but constitutes an interesting lever for plant adaptation to contrasted environmental conditions. All species used in cultivated grasslands are the same as those of natural grasslands. Therefore, studies on the genetic diversity in cultivated cultivars can be used to study processes in both natural and cultivated grasslands. Furthermore, recent studies by Blanco-Pastor et al. (2019, 2021) and Keep et al. (2020) show that the same mechanisms lay behind the natural diversity of *Lolium perenne*, a major component of both natural and sown grasslands, and those of registered varieties. The French Variety and Seed Study and Control Group (GEVES) conducts multi-year and multi-site experimentations to evaluate the performance of new cultivars^2^, therefore constituting the only database for assessing the relative contributions of genetic and environmental factors to the phenological diversity within a species (Figure 1). The analysis of 50 *Lolium perenne* cultivars from the French forage seed catalog revealed high genetic diversity in heading date with differences of up to 45 days between the earliest and latest cultivars, all sites and years considered (Figure 2). The intraspecific variability in heading date is similar to that observed between sites and years, thus constituting an interesting pool of genetic resources to increase diversity and durability of grasslands (Prieto et al., 2015; Litrico et al., 2016). Lastly, models of reproductive development should lead to better predictions of the dynamics of biomass quantity and quality in order to optimize grassland management.

Given the diversity of disciplines involved in the study of phenology (from genomics to ecology), it is important to determine the level of organization to be represented according to the objectives of the model. Agronomic indicators such as biomass quantity, quality and perenniality of grassland result from the cumulative behavior of the individual plants constituting the canopy. Furthermore, each perennial grass plant is a collection of tillers which have very different developments (some tillers being reproductive while others remain vegetative). These differences ensue from the different ages and phenological stages of apices. In the literature, quantitative relationships between phenological processes and environmental conditions have mainly been established at three organization levels: canopy level, plant level and apex level. These levels of organization are suitable for studying the interactions between morphogenesis and phenology, which are often neglected in phenological studies. Hormonal signaling and gene expression take place at lower levels of organization, their role on the reproductive development was reviewed by Ionescu et al. (2017) and Wang and Forster (2017). Nevertheless, the current knowledge of these levels does not allow us to infer quantitative relationships between environmental condition and plant phenotype. This review, therefore, focuses on the processes involved in the reproductive development of grasslands at canopy, plant and apex levels. The aim of the present paper is to provide a synthesis of the different aspects of the reproductive development at plant and apex level that should be accounted for in models in order to improve the prediction of grassland functioning and thereby management. First, we review the current knowledge of the effects of the genetic × environmental interactions on the transition from a vegetative to a reproductive tiller and the impact on tiller development and morphogenesis. Then, we focus on the effects of reproductive development at whole plant scale *i.e*., on the interactions between the different tillers of a plant and the impact on plant perenniality, defined as the maintenance of a minimum tiller density following cutting. Finally, we give a critical review of the current simulation models of grasslands, detailing the assumptions related to the reproductive development. For the sake of clarity, the main stages of grass phenology and their botanical structure are defined and described in Supplementary Data 1.

## Floral Induction and Reproductive Development at Tiller Scale

In grasses, the tiller is a functional unit having its own shoot organs and root system. Also, all stages of reproductive development, from floral induction to seed production, occur at individual tiller scale (Supplementary Data 1). Below, we report the current knowledge of floral induction and its impact on the tiller development and morphogenesis.

### Inductive Conditions

The floral induction of a tiller leads to the transition from a vegetative to a reproductive apex. Without completion of the floral induction, tillers remain vegetative and produce vegetative phytomers indefinitely. Heide (1994) wrote a comprehensive review of the environmental conditions that induce floral transition in diverse perennial grasses. For a large majority of perennial grasses, floral induction can be divided into two successive phases referred to as “primary induction” and “secondary induction” which are mainly dependent on two environmental variables: temperature and photoperiod (Heide, 1994). In a majority of species, the distinction of two phases (dual induction), instead of a single continuous phase (single induction), is explained by the fact that tiller induction requires exposure to two different scales of the same environmental variable, *e.g.*, low and high temperature or short and long photoperiods.

The primary induction is a progressive phenomenon mainly controlled by low temperatures and short photoperiods, which corresponds to winter conditions in temperate regions. The completion of the primary induction depends on the interaction between temperatures, photoperiod and exposure duration of several weeks. In most perennial grasses, low temperatures (0–6°C) for a period of 4 to 20 weeks is sufficient to complete primary induction, whatever the photoperiod. Under higher temperatures, the influence of the photoperiod becomes significant and primary induction can only occur under short photoperiod conditions (Lindsey and Peterson, 1964; Heide, 1994; Aamlid et al., 2000). In some cultivars, however, low temperature or short photoperiod can be sufficient, whatever the value of the other climatic variable (Heide, 1987, 1988). In a majority of cases, the completion of primary induction is definitive, but in only a few cases detailed in Heide (1988). Within a given species, primary induction varies between populations in terms of temperature, photoperiod and exposure duration according to their genetic make-up which can depend on their geographical origin. In *Lolium perenne*, which has a large latitudinal extension (from Scandinavia to the European Mediterranean area), the range of conditions for primary induction varies from no-requirement for Mediterranean genotypes to a primary induction with necessary low temperatures enhanced by short photoperiods for more northern genotypes (6°C/8 h for over 3 weeks) (Aamlid et al., 2000). For various grass species, the apparent absence of primary induction was generally reported in ecotypes originating from regions with low risk of late frost and an early dry season (Cooper, 1960; Aamlid et al., 2000).

The secondary induction starts only once primary induction has been completed and requires exposure to long photoperiods over a generally short period (> 12 h for less than one week) (Evans, 1958; Heide, 1987; Aamlid et al., 2000). The effects of long photoperiods can be mimicked by an interruption of the dark period with exposure to light for less than 2 h around middle of the night (Heide et al., 1985). It seems that secondary induction is not related to an increase in the incident radiative energy (Cooper, 1958; Aamlid et al., 2000). As for primary induction, the critical photoperiod required varies with the geographical origin of the ecotypes. Genotypes from high latitude areas require the longest photoperiod and the greatest number of long photoperiod cycles. Within the species *Lolium perenne*, Aamlid et al. (2000) found that the critical photoperiod to obtain 50% of heading plants ranged from 12 h for a Mediterranean cultivar to ca. 17 h for the northern ecotypes. For most *Lolium perenne* genotypes, elevated temperatures have a positive effect on the completion of the secondary induction, implying that they could increase the completion rate of the secondary induction. For example, 8 days of exposure to long days (24 h) at 12°C are required to reach 50% of heading plants, while the same result is observed with only 4 days at 18°C (Aamlid et al., 2000).

Previous studies on different ecotypes of *Lolium perenne* (Aamlid et al., 2000) and *Festuca* (Bean, 1970) highlighted that both single induction and dual induction could exist within a single species, depending on the genetic make-up which can depend on the geographical origin of the population. What is more, the cross between different ecotypes is genetically possible and can result in a high variability in primary induction requirements, which questions the conceptual frontier between the single and dual induction cases. Similarly, hybrids of close species in the *Lolium* genus (*L. perenne*, *L. multiflorum*, *L. rigidum* and *L. temulentum*) have all intermediary induction requirements without clear distinction between single and dual induction (Cooper, 1960; Evans, 1960a). There is another example in *Phleum* where the wildtype *Phleum alpinum* has a dual induction while the cultivated hexaploid *Phleum* only requires an induction by long photoperiods, without primary induction (Heide, 1994).

As mentioned by Heide (1994), growing plants in various controlled conditions and observing floral transition or heading is a convenient methodology to determine the threshold requirements for floral induction. However, these results alone do not allow an exhaustive quantification of floral induction requirements. In particular, they do not allow discretization of floral induction advancement at a daily time step. Such information would be useful for the prediction of the floral transition under field conditions, where temperature and photoperiod vary from day to day.

### Perception and Integration of the Inductive Signals

If exposure to sufficient inductive conditions is necessary for a plant to become reproductive, the success of the floral induction also requires the plant to be competent to perceive and integrate inductive signals (Lang, 1965). In many cases, young plants remain unable to complete floral induction, staying in a so-called juvenile stage (Heide, 1994). The duration of this stage varies considerably between species and genotypes (60 to 110 days), whereas no explicit juvenile stage was reported in *Lolium perenne*, as plants can be primary induced as soon as seed germination starts (Cooper, 1960; Bommer, 1961). In contrast, the floral induction of seeds in *Dactylis glomerata*, *Festuca pratensis*, *Festuca rubra* and *Poa pratensis* was never observed even after long exposure to low temperatures (2°C for 116 days) in the dark (Bommer, 1961). The perception site of low temperature has not been determined to our knowledge, although apices can complete primary induction without any other organ, as shown by Arumuganathan et al. (1991) who obtained flowering plants of *Lolium temulentum* from excised shoot meristems exposed to low temperatures (2°C). In seeds, the apex sensitivity to low inductive temperatures depends on carbohydrate supply and could therefore be linked to the size of caryopses (Heide, 1994). Nevertheless, this dependence disappears after germination, as no relations were observed between the carbohydrate status of the apices in seedlings and their ability to be induced (King and Evans, 1991). The methodologies used to study primary induction always use long exposure periods to a constant temperature. Therefore, the integration time lapse for the inductive thermal signal by the plant still remains unknown. This review will not consider in detail the recent findings at molecular scale. However, it should be mentioned here that it has been shown that the expression of a limited number of genes *i.e., VRN1*, *VRN2* and *MADS-box* genes play a key role in the genetic variations of the primary induction (Colasanti and Coneva, 2009; Seppänen et al., 2010; Ergon et al., 2013; Wang and Forster, 2017).

The photoperiodic signal is perceived by leaves through specific photoreceptors. The use of mutants in the model grass *Brachypodium distachyon*, revealed that the initial perception of long photoperiods occurs through *phytochrome C* (Woods et al., 2014; Arojju et al., 2016). Similar results concerning the key role of the *phytochrome C* were observed in wheat for which Chen et al. (2014) proposed a theoretical model to explain the effects of the *phytochrome C* in the long-photoperiod induction process, depending on daylength and circadian clock (Chen et al., 2014; Song et al., 2015). The transmission of the photoperiodic signal to the apex has long been debated in the literature. King and Evans (1991) showed in *Lolium temulentum* that an increase in sucrose content at the apex was not necessary to trigger the floral transition, although inflorescence development is an important carbohydrate sink thereafter. Using contrasting photoperiod exposure on intact and defoliated plants of *Lolium temulentum*, Périlleux and Bernier (1997) confirmed that the signal was probably not sucrose although it originated from leaves. Finally, more recent studies suggested that the floral signal perceived at the apex is hormonal with a preponderant role of gibberellins in interaction with the protein FT (for a review see King, 2012).

### Apex Morphology and Functioning Following Floral Transition

Floral transition occurs when all steps in floral induction are achieved, leading to visible changes in apex morphology (Supplementary Figure 1.1.A). The earliest changes occur within the meristematic zone, shortly after the completion of the floral induction. In *Lolium perenne*, Gonthier and Francis (1989) observed no modification of the apex length (between 100 and 300 μm) when the primary induction was reached, and the mitotic index was similar to that of non-induced tillers (< 4%). Primary-induced apices kept the same morphological appearance if they were held under short photoperiods (8 h), even under high temperature (18°C). A significant increase of the meristem length (up to 700 μm) and of the mitotic index was observed for tillers exposed to long-photoperiod (20 h of light for eight days) after the completion of the primary induction (Gonthier and Francis, 1989). Apex length and mitotic index can be related to the initiation rate of primordium. Before the completion of the primary induction the number of leaf primordia remains approximately constant at the apex (Kemp et al., 1989). When the photoperiod is sufficiently long for the onset of the secondary induction, the number of primordia on the apex quickly increases due to a drastic reduction of the plastochron (time between the production of two successive primordia), while the phyllochron (time between the emergence of two successive leaves) remains almost constant (Malvoisin, 1984). During the secondary induction in *Lolium perenne*, the plastochron is divided by 3 to 11 depending on the cultivars leading to a large accumulation of primordia at the apex (Kemp et al., 1989). The date of increase in the rate of primordia production showed a high genetic variability among *Lolium perenne* cultivars, but was not correlated with the heading date (Cooper, 1950; Kemp et al., 1989; Hazard et al., 1996). Also in *Lolium perenne*, Gonthier and Francis (1989) observed that the accumulation of primordia was dependent on the number of long photoperiod cycles perceived by the tiller after the completion of the primary induction.

During the phases of apex lengthening and rapid primordium initiation, the first macroscopic marks of the floral transition appear on the apex. First, white stripes appear at the basis of the newly produced primordia. The stripes are characterized by a high cellular density and will later elongate into long internodes. Then, the morphology of the youngest primordia changes: a second ridge appears and will later become a spikelet, while the older primordia will differentiate into leaves. The mechanisms regulating the development of primordia during the floral transition into leaves or spikelets are largely unknown. Finally, the meristematic dome located at the top of the apex differentiates into the terminal spikelet, stopping new spikelet production (Malvoisin, 1984). The cessation of spikelet primordia production appears to be coordinated with the emergence of the flag leaf tip in *Lolium perenne* and wheat (Kemp et al., 1989). The accumulation of primordia during the apex differentiation defines the final number of leaves and the number of spikelets of the tiller. The number of leaves remaining to emerge and the size of the spike determine the heading date of the tiller. Some authors proposed to approximate the heading date from the final number of leaves assuming a constant phyllochron (Cooper, 1960).

### Leaf Appearance and Growth Rates

The number of leaves produced from tiller emergence to flowering varies between tillers. In *Panicum virgatum*, Van Esbroeck et al. (1997) found that late summer emerging tillers produced less leaves before heading (about seven) than those emerged earlier during spring (nine to eleven leaves). A similar observation was made in *Phleum pratense*, where the final leaf number of reproductive tillers was clearly related to their initiation date, decreasing from ∼ 20 leaves for the main tillers to 7 leaves for tillers initiated later in spring (Langer, 1956). For the genus *Lolium*, Cooper (1950) considered that the rate of leaf emergence was identical for all tillers throughout their vegetative development as well as throughout the primary induction, meaning that the final leaf number of each tiller was determined between the start of the secondary induction and heading. He also showed that the number of leaves produced from the start of the secondary induction was negatively correlated to the number of short photoperiod cycles perceived after the end of the primary induction.

In temperate regions, the beginning of spring is associated with a strong increase of grassland production related to an increase of leaf growth rate (multiplied by 3 at 15°C) (Barre et al., 2015). This event occurs at the same period as the floral induction, which therefore raises the question of a causal relation between the phenological state of the plant and the rate of leaf growth. Parsons and Robson (1980) showed that the phenomenon was due to an increase in potential leaf growth rate, meaning that the intrinsic response of leaf growth to temperature was modified. They also concluded that the acceleration of leaf growth occurred only for plants that had previously experienced winter conditions and that the change in the leaf growth rate was due to floral transition. In contrast, many authors stated that the photoperiod signal itself has a morphogenetic effect on leaf growth in both vegetative and reproductive developments (for a review see Hay, 1990) and this, independently of trophic status In studies on *Lolium perenne*, *Dactylis glomerata* and *Festuca*, Ryle (1966) observed that increased photoperiod (from 8 h to 16 h) caused an increase in the final length of both lamina and sheaths, which resulted from an increase of the leaf elongation rate. The rapid elongation rate was later related to an increase in cell division and elongation by Wu et al. (2004). Davies (1971) managed to experimentally decorrelate flowering and leaf growth rate, as she observed an increase of the leaf growth rate in tillers, which, while exposed to long photoperiods, did not further exhibit any reproductive development. These results are in line with the study of Hazard et al. (2006) which observed an increase of leaf growth rate at the same time in two *Lolium perenne* morphotypes with contrasted dates of double ridge stage and heading. Altogether, these results suggest that the increase in leaf growth rate observed in spring is most certainly a direct response to photoperiod, which simultaneously triggers the floral transition of primary-induced apices. In addition, the decorrelation between spring growth and reproductive development has been intensively used by breeders to increase the flexibility of grassland use by selecting plants with an early vegetative growth in association to a late heading (Sampoux et al., 2011).

In grass tillers, the final length of leaves usually increases from the basis of the tiller, and then decreases for the last ranks in reproductive tillers (Borrill, 1959; Van Esbroeck et al., 1997). For *Lolium temulentum* grown under natural conditions, the inflorescence initiation is concomitant with the elongation of the leaf which has the longest lamina, while sheaths become progressively longer up to the flag leaf (Borrill, 1959). In *Lolium perenne* and *Dactylis glomerata*, the elongation of leaves supported by long internodes, i.e., the last leaves of the tiller, ends only a few days before spike emergence (Davies, 1978).

### Internode Elongation

On vegetative tillers, internodes remain very short and generally do not elongate further, except in stoloniferous species. On reproductive tillers, a series of long internodes are produced, the last one before the first flower constituting the peduncle. This leads to a significant elevation of the canopy (Figure 1). Internode elongation of reproductive tillers has strong impacts on plant functioning and grassland management. Firstly, forage quality in late spring decreases as the proportion of internode increases in the total aerial biomass (Buxton and Marten, 1989; Chapman et al., 2014). Secondly, internode elongation increases the height of the terminal apices above ground, therefore increasing the exposure to climatic hazards (frost) and herbivores. In addition, internode elongation has strong impacts on the sink-source relations of the whole plant as internodes constitute the main net importers of carbon assimilates in the tiller. As a consequence, (i) assimilate allocation to roots is significantly decreased (Parsons and Robson, 1981) and (ii) the transfer of assimilates from reproductive to vegetative tillers decreases, which could lead to the regression of young vegetative tillers (Gillet and Breisch, 1982; Colvill and Marshall, 1984; Matthew et al., 2000). Later in the tiller development, carbohydrates from internodes will be remobilized toward the seeds (Clemence and Hebblethwaite, 1984; Barillot et al., 2016).

**FIGURE 3:**
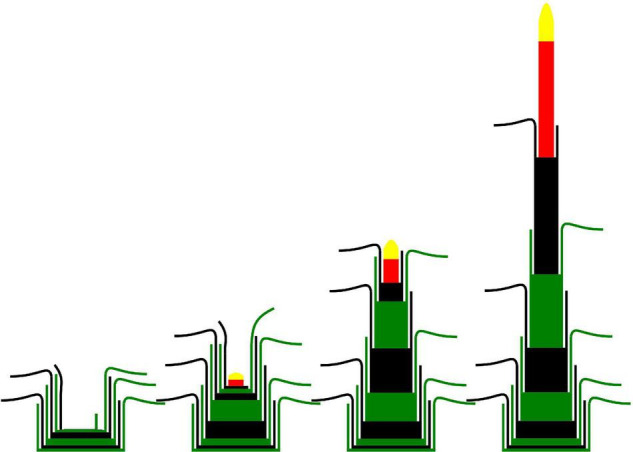
Schematic representation of leaf appearance, internode elongation and heading of a reproductive tiller (adapted from Gillet, 1980). Green and black phytomers represent successive phytomers. Red internode is the peduncle. Yellow structure is the inflorescence.

A majority of studies involving plant morphological measurements on perennial grasses do not differentiate the internodes composing the culm. However, as observed by Gillet (1980), the successive mature internodes have different final lengths and their elongation is not synchronous (Figure 3). The number of long internodes varies between species, cultivars and even the tillers of the same plant (4 to 6 in *Lolium perenne* and *Dactylis glomerata*) (Johnston and Waite, 1965). The total length of the culm was shown to vary with the emergence date of the tiller. In *Lolium perenne*, Aamlid et al. (2000) observed that culms were up to 10% shorter (peduncle included) for tillers emerged during the secondary induction compared to those emerged before or during the primary induction. In contrast, the duration of exposure to low temperatures (primary induction) had no effect on the final culm elongation in that study. These results are in accordance with the observation of shorter internodes in reproductive tillers developed during aftermath heading, in comparison with those developed during spring (Aamlid et al., 2000).

The timing of successive elongation of internodes has not been extensively studied in perennial grasses as it has been for wheat or maize, for which some coordination was established between internode and leaf elongation (Kirby et al., 1994; Fournier and Andrieu, 2000; Vidal and Andrieu, 2009; Zhu et al., 2014; Gauthier et al., 2020).

### Inflorescence Development

While the inflorescence of perennial grasses is considered of little interest for forage production, its development and morphology are of great importance for seed production (Scotton, 2018) and grassland ecology. In addition, inflorescence development plays an essential role in heading, as its relative size compared to the length of the flag leaf sheath. Perennial grass species exhibit a wide diversity of inflorescence morphologies, mainly spikes (*Lolium*, *Festuca*, *Bromus*, *Hilaria*, *Microchloa*), racemes (*Brachypodium*, *Pleuropogon*) and panicles (*Poa*, *Dactylis*, *Andropogon*) (Allred, 1982). These diversified morphologies depend on the branching intensity and duration of terminal and lateral meristems before spikelet formation (Perreta et al., 2009). In addition, the elongation of the inflorescence internodes affects the complete formation of the inflorescence and also determines the grass inflorescence morphology (Perreta et al., 2009).

Within species, inflorescences have the same general pattern but present slight differences between genotypes in terms of length, number of spikelets and number of flowers per spikelet. For instance, Aamlid et al. (2000) observed variations in the inflorescence (peduncle not included) among genotypes of *Lolium perenne*, which may be related to their geographical origin, as inflorescence length tended to increase with the latitude of origin. Byrne et al. (2009) found QTL (quantitative trait loci) involved in both inflorescence traits and heading date. Within *Lolium perenne* genotypes, Byrne et al. (2009) found a negative correlation between the heading date and inflorescence length, similar to what was observed in wheat (Donmez et al., 2001), whereas McGrath et al. (2010) reported a positive correlation.

Environmental conditions are also responsible for differences in inflorescence morphology, as observed among cultivars and tillers of a plant. In *Lolium perenne*, Colvill and Marshall (1984) found that tillers initiated early in the year preceding heading, i.e., during the previous summer, developed slightly longer inflorescences and had one or two supplementary spikelets than tillers produced later during autumn and winter. Early tillers had 35% more florets by spikelet compared to late tillers. Regarding the number of spikelets produced by the apex, Anslow (1963) showed for the *Lolium perenne* cultivar S24 that the number of spikelets was higher for late heading tillers. Under controlled conditions, it was determined for *Lolium perenne* and *Phleum pratense* that “short long photoperiods” and relatively low temperature (12h/13°C) during secondary induction decreased the final number of spikelets (Ryle and Langer, 1963; Ryle, 1965). Accordingly, Langer (1956) found in *Phleum pratense* that the later the tiller appeared, the shorter the ear length. In *Lolium perenne*, Aamlid et al. (2000) reported that tillers which emerged during the secondary induction developed shorter ears with fewer spikelets and florets than tillers emerged before or during the primary induction, and they also headed later. Evans (1960b) observed the production of abnormal inflorescences as the delay between the primary induction and the exposure to long photoperiods increased (secondary induction). Kleinendorst (1974) found a linear relation between the number of primordia at the beginning of the double-ridge stage and the final number of spikelets. The longer the period between primary induction completion and double-ridge stage, the longer was the apex at double-ridge stage and the higher the number of spikelets per ear.

## Sexual Reproduction Versus Vegetative Reproduction at Plant Scale and Impact on Tiller Demography

### Seasonal Evolution of Tiller Demography

**FIGURE 4:**
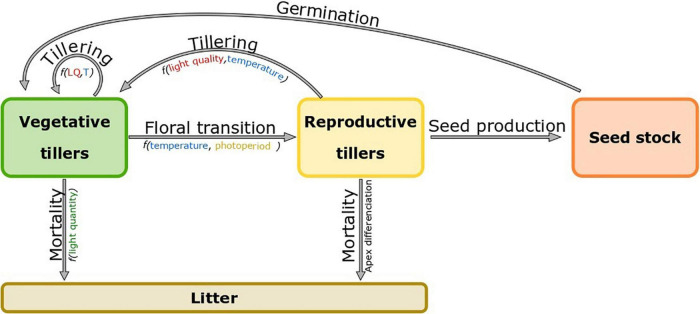
Compartmental model of a perennial grass plant. The plant is divided into three compartments: vegetative tillers (green), reproductive tillers (yellow) and dead tillers composing a litter (brown).

The pattern of tiller demography is usually similar in all temperate grasses (Darwinkel, 1978; Lecarpentier et al., 2019). First, from the sowing date the number of tillers rapidly increases up to a plateau (7000 tillers/m^2^ in *Lolium perenne* and 6000 tillers/m^2^ in *Lolium multiflorum*), which is usually reached at canopy closure (LAI > 3, with LAI the Leaf Area Index) (Simon and Lemaire, 1987). During spring and early summer, the number of tillers decreases due to the death, also called regression, of vegetative tillers (Figure 4). Synchronicity between vegetative tiller mortality and the reproductive phenology was regularly observed in *Lolium perenne* (cv. S23) (Ong et al., 1978; Colvill and Marshall, 1984), *Phleum pratense* and *Festuca pratensis* (Langer et al., 1964). Tiller regression usually occurs during the elongation of reproductive tillers and can reduce tiller population by 50% (Colvill and Marshall, 1984). Regressing tillers are mainly vegetative young tillers (age < 40 days), which experience an increasing competition for light and assimilates with larger and taller reproductive tillers (Ong, 1978; Colvill and Marshall, 1984; Sachs et al., 1993). After summer, reproductive tillers die after seed dispersion and the remaining vegetative tillers start to produce new leaves and tillers again until canopy closure is reached once again during the next growing season (Figure 4; Jewiss, 1972; Matthew et al., 2000). In addition, mowing or grazing can cause tiller death but also reduces the leaf area index (LAI) which in turn enhances tillering. Individual plant perenniality is enabled by a rapid turnover of successive tillers (Colvill and Marshall, 1984). Depending on the growth conditions experienced by individual tillers, part of them reach floral transition and become reproductive, while others remain vegetative (Figure 4). On the one hand, reproductive tillers decrease the overall number of tillers but determine the number of seeds produced and therefore the potential size of the next generation. On the other hand, the number of tillers which remain vegetative determines the future ability of a plant for resource capture.

In *Phleum pratense*, almost all tillers initiated before summer are reproductive, whatever the appearance date. Therefore, new vegetative tillers produced at the basis of reproductive tillers only emerge after the reproductive period. This mode of tiller replacement was qualified by Matthew et al. (2000) as “reproductive” pathway. In *Festuca pratensis*, Matthew et al. (2000) observed a long period of coexistence between reproductive and vegetative tillers, the former being replaced by tillers which emerged during spring and remained vegetative until the next spring. *Lolium multiflorum* has an important mortality of young tillers in spring in relation to the specificity of the floral induction in this species which does not necessarily require primary induction. In *Lolium perenne*, tiller emergence follows an approximately permanent regime throughout the year (Matthew, 1992). Thus, many age classes of tillers coexist in this species where the youngest ones ensure tiller replacement, following a so-called “vegetative” pathway.

### Proportion of Reproductive Tillers in Interaction With the Environmental Conditions

In the study of Barre et al. (2017), natural populations of *Lolium perenne* originating from all Europe were cultivated in two common garden experiments located in Lusignan (France) and Melle (Belgium). The authors found in both locations that the proportion of reproductive tillers was always higher in populations originating from low latitudes, which are characterized by a large diurnal temperature range and hot temperatures during the warmest months. In contrast, populations from oceanic climates had the lowest proportion of reproductive tillers. These results suggest that the adaptation of species and populations to their environment led to a higher production of seeds in regions where dry and hot summers alter the vegetative growth. On the contrary, the proportion of reproductive tillers is lower in populations from temperate regions (Poirier et al., 2012), where summer conditions allow some significant vegetative growth. In addition, the proportion of reproductive tillers per plant was found to be highly variable between natural populations of *Lolium perenne*, ranging from 13 to 72% (Barre et al., 2017).

Obviously, the proportion of reproductive tillers is also controlled by the environmental variables responsible for floral induction. For instance, low temperatures during primary induction as well as short photoperiods increased the number of reproductive tillers in *Lolium perenne* plants cultivated in growth chambers (Aamlid et al., 2000). Similarly, the reproductive tiller proportion was increased by the exposure to long photoperiods during the secondary induction (Aamlid et al., 2000). These observations emphasize that reproductive tiller proportion and floral induction present the same pattern of response to the inductive conditions. Therefore, the earliest cultivars, characterized by lower induction requirements in duration and intensity, produce the highest number of reproductive tillers. While nitrogen fertilization is known to have a positive effect on tillering in grasses, Bahmani et al. (2002) showed in *Lolium perenne* that the reproductive tiller proportion was higher in “Ellett” than in “Grasslands Ruanui” cultivar at low nitrogen fertilization. However, increasing nitrogen availability increased the reproductive tiller proportion in “Ellett” but not in “Grasslands Ruanui”. Conversely, the authors found no effect of water availability on the reproductive tiller proportion. Furthermore, Casal et al. (1985) demonstrated that light quality has an impact on reproductive tiller proportion, by manipulating the R/FR ratio perceived by tillers. In that experiment, the number of flowering tillers in a reproductive plant of *Lolium multiflorum* increased from 2 to 2.7 under low R/FR. However, an opposite response was observed in the two *Lolium perenne* cultivars “Ellett” and “Grasslands Ruanui” (Bahmani, 2000). This may highlight two different aptitudes to maintain vegetative tillers in dense canopies which are characterized by low R/FR (Casal et al., 1986). Bahmani et al. (2002) also observed that despite similar heading dates, the two cultivars of *Lolium perenne* “Ellett” and “Grasslands Ruanui” presented large differences in reproductive tiller proportion.

Defoliation by grazing or cutting also affects the reproductive tiller proportion. First, defoliation changes the light environment perceived by the axillary buds located at the basis of the plant, which can therefore trigger their development into new tillers. When defoliation occurs below the apex of the flowering tillers, which may even occur before heading, the tillers die. After cutting in late spring, a majority of the remaining tillers are vegetative. For some species and cultivars, the cutting of flowering tillers marks the end of the flowering period until the next growing season. However, in some cases, a second wave of flowering starts with the development of new reproductive tillers during autumn. Named “aftermath heading,” this behavior is mostly represented in early-heading genotypes (Arojju et al., 2016).

### Inheritance of the Reproductive State From Mother Tillers

Some early researchers assumed that the floral transition occurred independently at the tiller scale i.e., each tiller had to experience the whole induction process (primary and secondary induction) to become reproductive (Kleinendorst, 1974). For species and genotypes in which primary induction can be completed under the only effect of temperature, no juvenile stage is observed, meaning that each apex is competent to be primary induced, whatever its initiation date. However, because the secondary induction requires the presence of leaves to perceive the photoperiodic signal, the floral transition of the primary induced apices will only be effective after the emergence of the first leaf of the tiller. This hypothesis is sufficient to explain the reproductive tiller proportion in some species such as *Lolium perenne*. However, some studies highlighted that the hypothesis of an independent induction process for each apex cannot explain the reproductive tiller proportion of species whose primary induction is partly enabled by the photoperiod. In *Bromus inermis* and *Dactylis glomerata* for instance, the primary induction is mainly controlled by short photoperiods, Havstad et al. (2004) observed that many tillers emerging after the end of the short photoperiod treatment were still able to become reproductive, in spite of having no leaf exposed to light during that stage. In another experiment, Havstad et al. (2003) exposed only half-plants of *Dactylis glomerata* and *Bromus inermis* to short photoperiods. Although these species have a necessary short-photoperiod requirement for primary induction, he observed flowering in non-exposed tillers. These observations led to the hypothesis of an inductive signal transferred from the reproductive to the vegetative tillers of the same plant (Havstad et al., 2003, 2004). However, the nature of such an indirect inductive signal remains to be determined to date and raises the question of the conservation of vegetative tillers in the plant if such an inductive signal exists. This would require the involvement of a competing floral inhibitor (Havstad et al., 2004). On the contrary, it was also reported that in some cases, the youngest tillers may experience floral transition while the oldest ones remain vegetative. For instance, Williamson (2008) observed in *Lolium perenne* that the first primary tiller was flowering in only 28% of plants whereas the second, third and fourth primary tillers were flowering in 94, 62, and 30% of plants, respectively.

## Modeling Grassland Phenology: What Is Known and What Is Still to Be Determined

Numerical models designed for grassland management seek to account for the reproductive development in order to better predict the seasonality of biomass production, as well as grassland perenniality. Although annual species can compose grasslands, the majority of species are perennial and they enable grassland perenniality through the production of new vegetative tillers able to survive the reproductive season of the grass. One of the difficulties encountered in modeling the phenology of grasslands is the representation of this perenniality and in particular, the presence of a large number of tillers of which only a part will undergo floral transition over a growing season. The models may also constitute research tools for anticipating the effects of climate change on grasslands and identifying plant ideotypes adapted to future growing conditions. Below, we present a review of existing models of grassland phenology, highlighting their respective objectives and assumptions (Table 1).

**TABLE 1:** The reproductive phenology in current grassland models.

ID	Name	Reference	Type	Species		Reproductive phenology				
						Primary induction	Secondary induction	Floral transition	Heading	Other
1	-	Fiorelli et al., 2001	Mechanistic model of tiller population	*Lolium perenne*		-	-	DOY* of first tiller conversion	Emergence of the 1^st^ complete inflorescence (DOY)	-
2	OSYAQ	Herrmann and Schachtel, 2001	Organ compartments	*Lolium multiflorum* for calibration and validation		-	-	-	-	Change of organ demand is dependent on a sum of daily development rate (*beta* function of temperature) from sowing date
3	CATIMO	Bonesmo and Bélanger, 2002	Organ compartments	*Phleum pratense*		-	-	Sum of GDD** basis 0 from May 1^st^***	Sum of GDD** basis 0 from May 1^st^***	-
4	OSYAQ	Herrmann and Schachtel, 2001	Organ compartments	*Lolium multiflorum* for calibration and validation		-	-	-	-	Change of organ demand is dependent on a sum of daily development rate (*beta* function of temperature) from sowing date
5	GrazeGro	Barrett et al., 2005	Crop model	*Lolium perenne*		-	-	Mean time of double ridge stage (input value)	Date (input value)	-
6	SISTAL	Mazel et al., 2005	Individual based	Perennial grass species but developed on *Festuca arundicea*		Tillers should be born before the end of winter	-	Probabilistic function of tiller birth date	-	-
7	ModVege	Jouven et al., 2006	Crop model	Several species		-	-	Sum of GDD from January 1^st^***	-	-
8	STICS grasslands	Jégo et al., 2013	Crop model	*Phleum pratense*		-	-	Sum of GDD** (provided by CATIMO model)	-	-
10	BASGRA	Höglind et al., 2016	Process-based model	Calibrated with *Phleum pratense*	Threshold temperature (low)		-	-	-	-
10	BASGRA_NZ	Woodward et al., 2020	Process-based model	*Lolium perenne*	Incremental function of the temperature		-	-	-	-

**ID**	**Tiller demography**				**Environmental/Managing factors considered for floral development**					
	**Proportion of reproductive tillers**	**Aftermath heading**	**Tillering**	**Tiller mortality**	**Genetics**	**Water availability**	**Mineral nutrition**	**Light radiations**	**Photoperiod/Latitude**	**Cutting/Regrowth**

1	Linear function of the tiller appearance date during a favorable period	Emergent property	Constant number of live tillers	Death of reproductive tillers when their height is above cutting height	Three cultivars differing in heading date		Effect of nitrogen on leaf growth	-	Latitude. Model validated for 2 latitudes 52°N and 67°N	Yes
2	-	-	-	-	Yes	Change of organ demand	Change of organ demand	Yes	-	Yes
3	-	-	-	-	Yes	Effect of drought on RUE	Effect of nitrogen on RUE	-	-	-
4	Linear function of the tiller appearance date during a favorable period	Emergent property	-	Death of reproductive tillers when their height is above cutting height	Three cultivars differing in heading date	-	Effect of nitrogen on leaf growth	-	Latitude. Model validated for 2 latitudes 52°N and 67°N	Yes
5	All tillers appeared before a given date will become reproductive (March 1^st^***). Timing of tiller headings follow a normal distribution around the input heading date	-	Affected by flowering	Death of reproductive tillers by decapitation	3 classes of precocity	Yes	Yes	-	-	Impact on leaf growth after flowering tiller decapitation
6	Tiller transition follow a function of tiller birth date. Tillers born after the end of November will remain vegetative. Tillers born before the end of August have the highest chance to become reproductive ***	-	Yes	Vegetative tillers	Yes	Yes	Yes	Canopy closure	-	Yes
7	Reproductive growth is represented by a function of nitrogen nutrition. Start and end- are GDD* sum from January 1^st^***	-	-	-	4 groups of species	Yes	Nitrogen	-	-	Cutting stops reproductive growth
8	-	-	-	-	-	-	-	-	-	-
9	Tillers become non-elongating reproductive at a daily rate depending on temperature and daylength. Conversion from non-elongating to elongating tiller category follow a constant daily rate if daylength remains above a minimum value	Emergent property	Vegetative tillers are produced proportionally leaf appearance, but site-filling is reduced when LAI is high or C reserves are low	By frost and decapitation	Yes	-	-	Yes	-	Yes
10	Tillers become non-elongating reproductive at a daily rate depending on temperature and daylength. Conversion from non-elongating to elongating tiller category follow a constant daily rate if daylength remains above a minimum value	Emergent property	Vegetative tillers are produced proportionally leaf appearance, but site-filling is reduced when LAI is high or C reserves are low	By frost and decapitation	Yes	-	-	Yes	-	Yes

* DOY: day of year.

** GDD: growing degree-day.

*** Calendar date fits for the northern hemisphere.

### Simulation of Floral Transition and Heading Date

In most empirical implementations of reproductive development, reproductive events occur at fixed dates expressed either in growing degree-days (GDD) or in calendar time. In the first case, the sum of GDD is initialized at a given date which was empirically determined, January 1st for the model ModVege (Jouven et al., 2006) or May 1st for model CATIMO (Bonesmo and Bélanger, 2002) and STICS (Jégo et al., 2013). Regarding the models based on calendar time, they need to be recalibrated for every location, in order to account for the effect of the latitude on the photoperiod. In addition, these models do not account for the effect of temperature on the growth of reproductive structures (internodes, spikes), which prevents them from being used for predicting flowering variability in response to interannual fluctuations of temperature or to climate change.

In models considering the grassland as a population of tillers, the primary induction of each tiller is assumed to occur in tillers emerged before a critical date, while others remain vegetative (Fiorelli et al., 2001; Groot and Lantinga, 2004; Barrett et al., 2005; Mazel et al., 2005; Jouven et al., 2006; Höglind et al., 2016). The calibration of these models is based on empirical relationships between tiller emergence date and floral transition, as observed in different locations and years. To the best of our knowledge, none of the grassland models accounts for the sequential nature of floral induction i.e., the progressive completion of primary and secondary induction. A recent version of the model BASGRA, named BASGRA_NZ, considers primary induction in more detail (Woodward et al., 2020). In this model, primary induction is incremental and calculated at canopy scale. The daily increment of primary induction is calculated by a concave function of the soil surface temperature and a calibrated parameter representing the optimal temperature for primary induction.

### Simulation of Reproductive Tiller Proportion and Its Impact on Plant Perenniality

Models CATIMO (Bonesmo and Bélanger, 2002) and OSYAQ (Herrmann and Schachtel, 2001) do not explicitly distinguish the functioning cycle of vegetative tillers from that of reproductive tillers. Variations in tiller demography are therefore implicit and the increase in reproductive tillers is derived from the decrease in the biomass quality for herbivores, as calibrated from biomass harvests performed throughout the season. Few models consider the role of environmental conditions in the proportion of reproductive tillers. In the model developed by Fiorelli et al. (2001), the proportion of reproductive tillers increases with nitrogen availability. In more detailed models, the population of tillers is distributed in groups according to their status, *e.g.*, vegetative and reproductive in the model of Groot and Lantinga (2004), GrazeGro (Barrett et al., 2005) and SISTAL (Mazel et al., 2005); vegetative, non-elongating reproductive and elongating reproductive in BASGRA (Höglind et al., 2016) and BASGRA_NZ (Woodward et al., 2020); or vegetative, reproductive and senescent in Fiorelli’s (2001). In the model of Fiorelli et al. (2001), the size of the tiller population is considered constant as new tillers permanently replace the reproductive ones. This is representative of the established swards but not of the early stages of sward development. For models which dynamically simulate tiller demography (GrazeGro, SISTAL and BASGRA), tiller appearance is dependent on the rhythm of leaf production and stops at canopy closure, approximated by using a leaf area index threshold. The transition of tillers from a developmental group to another is based on different hypotheses. In Groot and Lantinga (2004), initial tillers are undifferentiated, those which appeared before the end of the vernalization period become reproductive according to their order of emergence and a constant daily rate. In SISTAL (Mazel et al., 2005), the probability of any tiller becoming reproductive is defined by its date of initiation: tillers initiated before the end of November remain vegetative and those initiated before the end of August are the most likely to become reproductive. In the model GrazeGro (Barrett et al., 2005), all tillers which appeared before March 1st are considered to be primary induced and will therefore become reproductive. The model BASGRA (Höglind et al., 2016) simulates plant development for several years and is the only one which explicitly accounts for the frost-induced mortality of vegetative tillers. The reproductive transition of the tillers in BASGRA is more sequential; a first transition from vegetative to non-elongating reproductive tillers occurs after primary induction (temperature threshold) and depends on temperature and photoperiod, the subsequent transition in elongating reproductive tillers occurs at a constant daily rate if the photoperiod is sufficiently long. In the model BASGRA_NZ, the transition is similar to the model BASGRA, but primary induction is more complex. The primary induction of tillers occurs at a daily rate depending on the temperature.

Modeling the reproductive tiller proportion throughout the season also requires consideration of tiller mortality. Tiller mortality due to cutting is inconsistently implemented in grassland models. In GrazeGro (Barrett et al., 2005), all reproductive tillers are removed after cutting by grazing or mowing. In BASGRA (Höglind et al., 2016), which distinguishes two categories of reproductive tillers, the mortality by cutting only applies to elongating tillers, assuming that the apices of non-elongated tillers are located below the cutting point. In the model of Groot and Lantinga (2004), which has a geometrical representation of tillers, only the reproductive tillers whose apex is above the cutting height are removed. The model SISTAL (Mazel et al., 2005) is the only one to integrate the mortality of vegetative tillers occurring during the reproductive period. Aftermath heading is also accounted for in a few models which assume that some individual reproductive tillers may survive after mowing or grazing. In the model of Groot and Lantinga (2004), aftermath heading is an emergent property resulting from the height of the apex and the cutting height. In BASGRA (Höglind et al., 2016), reproductive tillers can remain non-elongated, thus avoiding mortality by cutting. Interestingly, some models also include some effects of winter, in particular freezing risks, which allow simulation of a complete annual cycle of the plant (Höglind et al., 2016).

### Toward More Comprehensive Models of Grassland Reproductive Phenology

Current models of grassland are mostly based on empirical rules with parameters established for given genotypes cultivated under specific environmental conditions. Therefore, the parameters of these models may be valid only in the calibration conditions. Also, the specific genetic and environmental components of the parameters cannot be easily distinguished. In order to further improve our ability to predict the phenology of grasses and its effects on grassland productivity and management, we propose to develop more comprehensive models integrating the role of the environment × genetic interactions on the reproductive phenology on the one hand and vegetative processes on the other hand at individual tiller scale. Such models should be more mechanistic, with the explicit parametrization of each kind of processes, all controlled by genetics and environmental conditions. We have seen that floral induction is a critical event for the onset of flowering which is highly dependent on genetic × environment interactions. In our opinion, a fine implementation of this process in models is essential to better anticipate the phenology of grasslands in current and future climates. However, it would require better knowledge of floral induction, in particular concerning the cardinal temperatures and photoperiods associated with the primary and secondary floral inductions of each species and genotype. The determination of induction equations independent of the experimental conditions would require the use of experiments under constant induction conditions combined with experiments under fluctuating conditions. Finally, a method for an early determination of floral transition would also be required (*e.g.*, phytohormone dosage, non-destructive observation of the apex) to replace the late determination of the reproductive state often observed at the date of heading.

Strong interactions between the reproductive development of perennial grasses and their morphogenesis are known and more evidence was highlighted in above chapters. These interactions are poorly accounted for in current models despite their impact on biomass quality and quantity. For instance, the rhythm of leaf appearance is an important variable for the determination of the heading date, as it determines the final number of leaves, the duration of their expansion and the length of the pseudostem from which the spike will emerge. Architectural models based on coordination rules between successive leaves (*e.g.*, Verdenal et al., 2008) would appropriately account for the temporal evolution and the intraspecific diversity of the rhythm of leaf appearance in relation with growing conditions. According to the studies reviewed above, the increase in leaf growth rate usually observed in spring should be implemented in models as a direct function of temperature and photoperiod i.e., independently of floral transition. Finally, the modeling of tiller appearance and its reproductive status is the key point to predict each tiller’s perception of environmental conditions, the number of spikes, the proportion of stem in the harvested biomass and the tiller demography. Therefore, it seems that the tiller is the relevant scale to model the reproductive development of perennial grasses. The individual-based models describing the tillering dynamics therefore appear as promising tools to better integrate the growth conditions actually experienced by individual tillers (Lafarge et al., 2005; Mazel et al., 2005; Verdenal et al., 2008; Rouet et al., 2020). In addition, the topological and geometrical relations between tillers represented in these models are adapted to further assess the hypothesis of a signaling system responsible for the transmission of floral induction between tillers. Individual-based models coupled with an explicit description of plant architecture in 3D also give the opportunity to determine precisely the environment perceived by each individual tiller, leaf and bud (Godin and Sinoquet, 2005). It also allows explicit representation of the heterogeneity of both plant structures and environment within the canopy, by attributing different genetic characteristics to the plant (DeJong et al., 2011).

## Conclusion

Agriculture is facing new issues, requiring more efficient and sustainable agrosystems based on species and cultivars adapted to the future climatic conditions. A more sensible management based on more powerful numerical applications is a key to the sustainability of future agriculture. In perennial grasses, these objectives strongly depend on the reproductive phenology, as the earliness and proportion of reproductive tillers affect spring production and grassland perenniality. Computer plant modeling is a promising tool to understand plant functioning under current environmental conditions, simulate changes due to future conditions and test genetic and management solutions. The accuracy of the models depends on the knowledge we have of the development of plants in interaction with their environment.

The present review highlights that two main scales have to be considered to fully address the reproductive development of the whole plant. Floral induction and the growth of reproductive organs mainly proceed at tiller scale. This is in accordance with the fact that morphogenesis (leaf production and elongation) and metabolism are highly independent between the tillers of a cohort (except in early stages). Floral induction depends on the environmental conditions and determines the morphogenesis and, at the same time, the heading date of the tiller. Accounting for the plant scale is also necessary to understand the tiller demography, from bud initiation to death. This review highlighted some points that would require further investigations, such as a better quantification of the inductive requirements for floral induction. For instance in some species and conditions, the extent to which the reproductive status of tillers could be inherited from other tillers of the plant should be better assessed and quantified.

Current models of perennial grasslands have been developed for agronomic use with different degree of detail. Nevertheless, these models do not allow us to simulate the dynamics of floral transition at tiller scale in perennial grasses, which limits our ability to study how genotype × environment interactions affect plant phenology. A rough representation of the tiller population along a whole year also limits our ability to study the perenniality of plants and the competition between them. As a further step, future models should better account for the dual floral induction process. So far, vegetative and reproductive development have often been studied separately, but the present review highlighted that their interaction should be considered. There is a lack in the representation of the interactions between reproductive development and morphogenesis considered at tiller and plant scale. Representing the whole plant scale is also crucial to account for processes determining the demography of tillers and therefore plant perenniality, among which tillering regulation, the coordination between leaf production and tiller initiation. Including such aspects in future models is essential to improve our ability to predict the reproductive phenology of grasslands in contrasted climatic conditions and management.

## Author Contributions

SR, RB, and J-LD contributed equally to the writing of the manuscript. All authors contributed to manuscript revision, read, and approved the submitted version.

## Conflict of Interest

The authors declare that the research was conducted in the absence of any commercial or financial relationships that could be construed as a potential conflict of interest.

## Publisher’s Note

All claims expressed in this article are solely those of the authors and do not necessarily represent those of their affiliated organizations, or those of the publisher, the editors and the reviewers. Any product that may be evaluated in this article, or claim that may be made by its manufacturer, is not guaranteed or endorsed by the publisher.

## Footnotes

1
http://www.fao.org/3/y8344e/y8344e05.htm

2
www.geves.fr
