# Supplementary material for: Interactions Between Environment and Genetic Diversity in Perennial Grass Phenology: A Review of Processes at Plant Scale and Modeling
Source: Front Plant Sci. 2021 Nov 16;12:672156. doi: 10.3389/fpls.2021.672156 (PMC8635016; doi:10.3389/fpls.2021.672156)
Supplement: Supplementary file 1 [file Data_Sheet_1.docx]

Supplementary Material

# Supplementary Data

## Botanical structure, vegetative and reproductive cycles in perennial grasses

In perennial grasses, individual plants consist in a colony of genetically identical cohorts of tillers, originating from the same seed generally sown at the beginning of spring or during autumn in temperate regions. At the tiller level, ***vegetative*** and ***reproductive*** development are successively realized by the same apex (Supp Figure 1). After seed **germination** (Supp Figure 1.1), a first vegetative ***tiller***, called main stem, emerges from the seed and grows (Supp Figure 1.2). All vegetative tillers can be represented as a succession of vegetative ***phytomers***, each initiated by the terminal apex and constituted of a ***leaf*** (lamina and sheath), an ***internode*** and a ***node*** which bears an ***axillary bud*** (Moore and Moser 1995). During the vegetative stages, the ***terminal apex*** (Supp Figure 1.A, 1.B) of a tiller is usually located close to the ground, inside the **pseudostem** made of the superposition of leaf sheaths (Thomas 1980). The apex bears several vegetative primordia which are emitted periodically (plastochron) and will later develop into a phytomer. New tillers originate from the axillary buds located at the axil of each leaf (Supp Figure 1.3) (Davies and Thomas 1983). The development of axillary buds into tillers, called **tillering** (Jewiss 1972; Thomas 1980), is under control of the environment, in particular the Red/Far-Red ratio (Casal et al. 1986, Gautier et al 1999, 1987; Casal 2013). Depending on the environmental conditions experienced by each tiller, ***floral induction*** may be completed (Supp Figure 1.3-1.5), thus leading to the ***floral transition*** of the apex, which then becomes reproductive (Supp Figure 1.C). This new status modifies the primordia appearance rate and leads to the elongation of non-mature internodes (Supp Figure 1.6) and finally the formation of ***spikelets*** (Supp Figure 1.D) (Gillet 1980). These reproductive phytomers are the last phytomers and form an ***inflorescence*** (Supp Figure 1.E). Not all tillers of a plant become reproductive, some remain vegetative *i.e.* their terminal apex keep producing vegetative phytomers (Supp Figure 1.5-1.7). For reproductive tillers, internode elongation leads to the progressive increase of apex and tiller height. At the end of ***flag leaf*** expansion and due to the elongation of the inflorescence and peduncle, the ***terminal spikelet*** appears out of the pseudo-stem. This event is referred to as the ***heading*** of the tiller (Supp Figure 1.6) (Gillet 1980; Thomas 1980). The further development of the inflorescence conducts to anthesis, seed formation and seed maturation (Supp Figure 1.7). After seed maturation, the tiller ***senescence*** is complete. ***Tiller death*** can also occur earlier if the apex is removed by mowing or grazing. Tillers remaining vegetative ensure ***plant perenniality*** through leaf production and tillering. Thus, two pathways coexist in perennial grasses to ensure plant survival: the sexual reproduction by ***seed production*** (Supp Figure 1.8) and the ***vegetative multiplication*** by tillering (Supp Figure 1.9) (Gillet 1980).

# Supplementary Figures and Tables

## Supplementary Figures


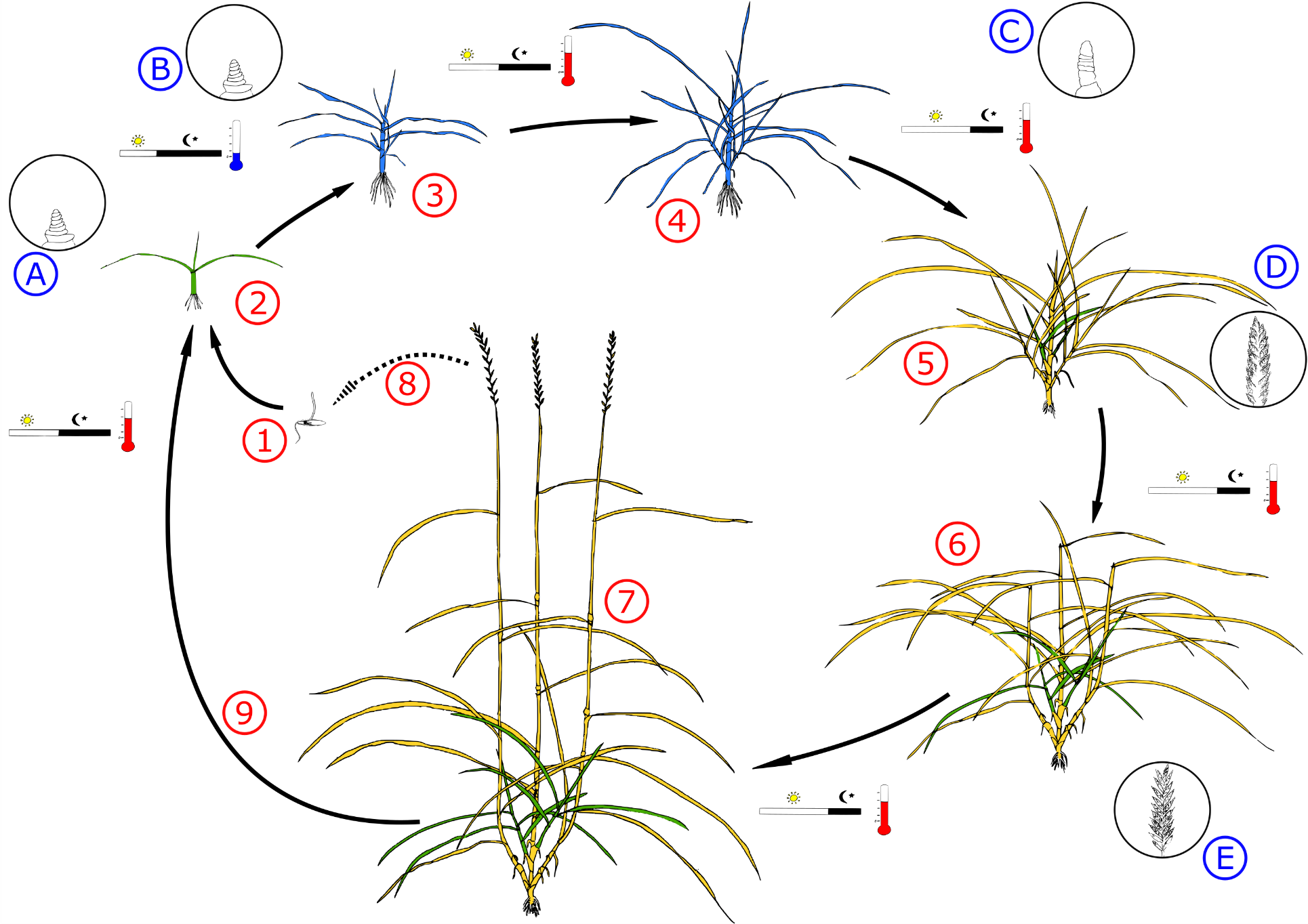


**Supplementary Figure 1.** Life cycle of a perennial grass plant and the evolution of apex morphology in relation with climatic conditions. Variations in photoperiod are represented by horizontal bars indicating the proportion of day and night within 24 hours. Three configurations are represented: day/night ≈ 1 (equivalent to spring and autumn), day/night > 1 (equivalent to summer), day/night <1 (equivalent to winter). Two temperature levels are represented: mean/elevated temperature (red thermometer) and low temperature (blue thermometer). The main steps of the vegetative and reproductive development are represented; **1**: Seed germination; **2**: Vegetative development of the main tiller; **3**: Plant ramification with the emergence of two tillers and primary induction of the three tillers (in blue); **4**: Increase of leaf growth rate producing longer leaves; **5**: Secondary induction of the first three present tillers (in yellow). Emergence of two supplementary tillers, which did not reach primary induction and remain vegetative (in green); **6**: Heading. Emergence of the last leaves of the reproductive tiller, start of internode elongation and emergence of the last spikelet from the pseudo-stem; **7**: Anthesis. End of internode elongation, flower development and stamina emergence; **8**: Completion of the reproductive pathway by seed production, after fecundation and seed filling; **9**: Completion of the vegetative pathway ensured by the tillers remaining vegetative; **A**: Apex of the main stem bearing primordia differentiating in leaves and undifferentiated primordia; **B**: Permanent regime. The rate of primordia emission by the meristematic area is equivalent to the rate of leaf emergence; **C**: ‘Double-ridges’ stage. Differentiation of primordia with the appearance of a second wrinkle; **D**: Spikelet development; E: Heading. Spike emerges from the last leaf sheath. Spikelet number of the spike is determined but internodes between spikelets are not elongated
